# Supplementary figures and images for: Quantitative Test of the Barrier Nucleosome Model for Statistical Positioning of Nucleosomes Up- and Downstream of Transcription Start Sites
Source: PLoS Comput Biol. 2010 Aug 19;6(8):e1000891. doi: 10.1371/journal.pcbi.1000891 (PMC2924246; doi:10.1371/journal.pcbi.1000891)

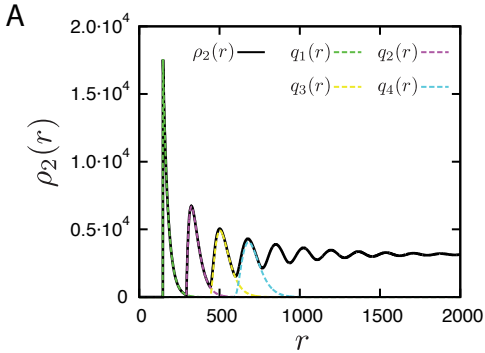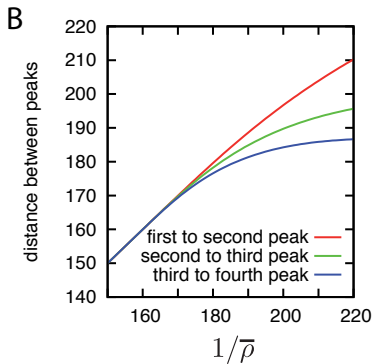

Supplement: Figure S1 — Characteristics of the Tonks gas two-particle distribution function. (A) Two-particle distribution function for a particle size of and an average particle spacing . The first few individual terms contributing to are superimposed. (B) Distance between the individual peaks in as a function of for . For dense packing, the first few maxima are equidistantly spaced by . Note that the first peak is always located at , regardless of the particle density. (0.32 MB PDF) [file pcbi.1000891.s001.pdf]

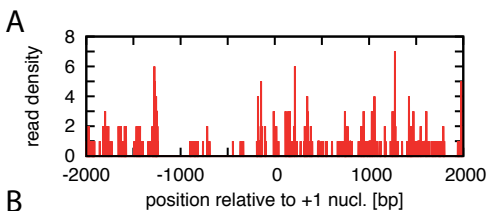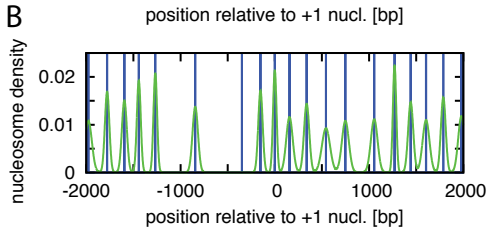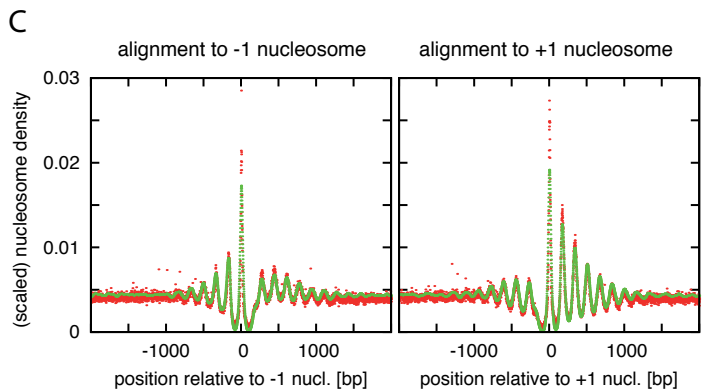

Supplement: Figure S2 — Comparison between two proxies for the nucleosome density. (A) Section of the read density map () based on sequence reads reported by Mavrich et al. [10]. (B) Section of nucleosome density estimate based on the list of nucleosomes identified by Mavrich et al. (): Each nucleosome is represented by a Gaussian with mean and standard deviation corresponding to the values reported. (C) Alignment of both nucleosome density proxies (red dots for , green dots for ) to nucleosome positions and averaging over all genes leads to nearly identical results. To account for the unknown normalization, we scaled the read density map such that the genome-wide number of reads equals the genome-wide number of identified nucleosomes. (0.46 MB PDF) [file pcbi.1000891.s002.pdf]

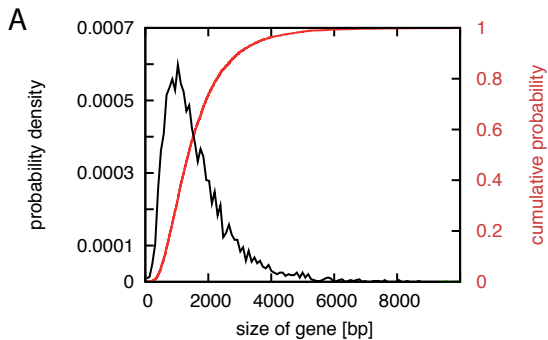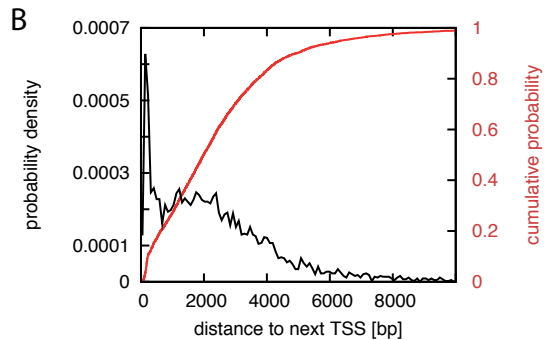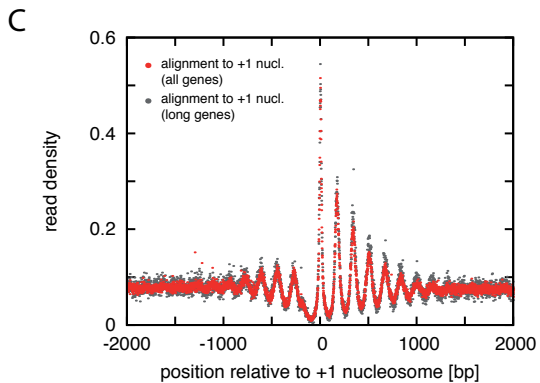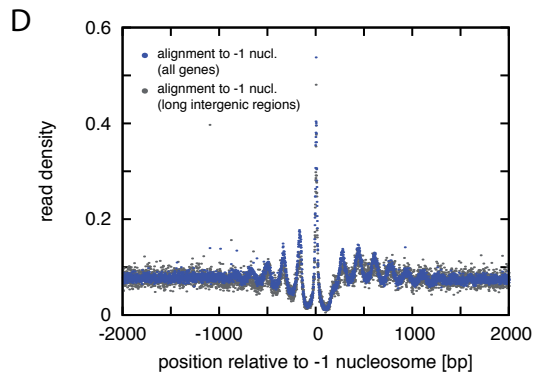

Supplement: Figure S3 — Distribution of gene start and end sites and effects on alignments. (A) Probability distribution (black) and cumulative distribution (red) for the length of genes. Typical sizes of genes are about 1000 bp, but about nearly a third is larger than 2000 bp. (B) Same distributions for the distance between neighboring TSSs. Distances are in general comparable to the size of genes, but a number of TSSs are very close to each other. (C) Alignment of read density to +1 nucleosome and average over all genes (red dots) and those 1269 genes being larger than 2000 bp only (gray dots). The averages are very similar, but close inspection shows that amplitudes are slightly larger and oscillations range further when considering large genes only. (D) Alignment of read density to −1 nucleosome and average over all genes (blue dots) and those 952 genes where no gene starts or ends were found within 1000 bp upstream of the TSS (gray dots). The averages are very similar, but amplitudes are slightly smaller when considering those genes without other gene starts or ends upstream only. (0.52 MB PDF) [file pcbi.1000891.s003.pdf]

read density

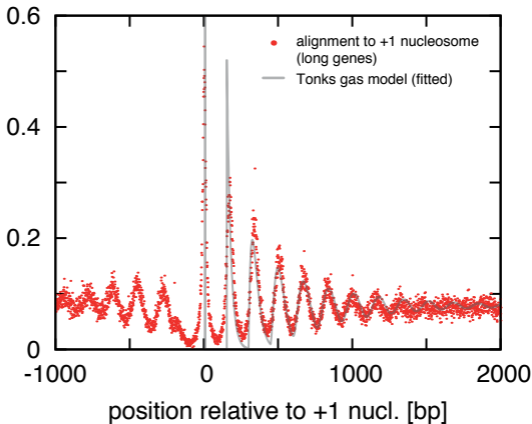

Supplement: Figure S4 — Best fit of Tonks gas model (gray line) to +1 nucleosome alignment of read density including genes larger than 2,000 bp only (red dots). Visual inspection yields good agreement between model and data, comparable to the analogous fit to the data including all genes (Fig. 2A, see also Fig. S3C). For estimated parameters see Table S1. (0.41 MB PDF) [file pcbi.1000891.s004.pdf]

A

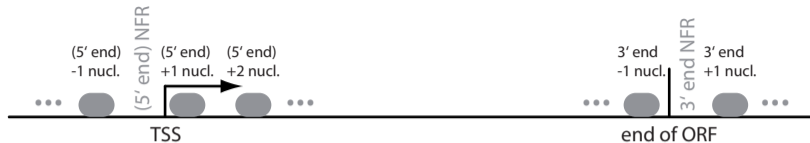

B

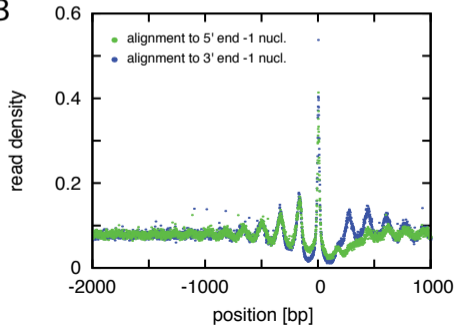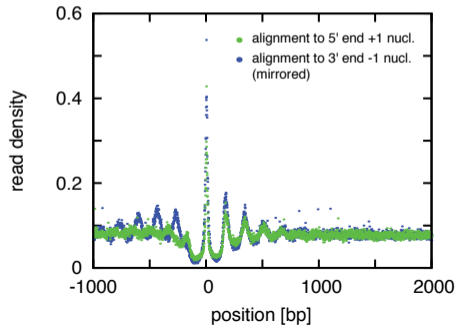

Supplement: Figure S6 — Nucleosome organization around the 3′ end of genes. (A) Sketch of a typical nucleosome organization around both the 5′ and 3′ ends of genes. Throughout this study, the focus is primarily on the 5′ NFR with its flanking −1 and +1 nucleosomes. The nucleosomes flanking the 3′ NFR are here referred to as the 3′ end −1 nucleosome and the 3′ end +1 nucleosome. We determined the positions of 3′ end nucleosomes in analogy to the 5′ end nucleosomes: The 3′ end −1 nucleosome is defined as the nucleosome at or first nucleosome upstream of the ORF end while the 3′ end +1 nucleosome is the first nucleosome downstream. (B) Alignment of read density to the 3′ end −1 nucleosome (left) and 3′ end +1 nucleosome (right), respectively (green data points). For comparison, the alignment to the 5′ end −1 nucleosome is also shown (blue data points, from Fig. 4, mirrored on the right). Overall, a good agreement is visible between the alignments to the nucleosomes flanking the 3′ NFR on both sides and the alignment to the 5′ end −1 nucleosome. This indicates that at the 3′ end the nucleosomes are only statistically positioned against a repulsive barrier, which we found to be the most likely scenario for the pattern upstream of the 5′ NFR. (Note the small bump in the read density within the nucleosome depleted region, just downstream of the 3′ end −1 nucleosome and upstream of the 3′ end +1 nucleosome; it indicates that the identification of 3′ NFRs is not perfect or a certain fraction of genes does not display a 3′ NFR.) (0.42 MB PDF) [file pcbi.1000891.s006.pdf]
