# Supplementary material for: Quantitative Test of the Barrier Nucleosome Model for Statistical Positioning of Nucleosomes Up- and Downstream of Transcription Start Sites
Source: PLoS Comput Biol. 2010 Aug 19;6(8):e1000891. doi: 10.1371/journal.pcbi.1000891 (PMC2924246; doi:10.1371/journal.pcbi.1000891)
Supplement: Figure S5 — Best simultaneous fits (gray lines) to −1 and +1 nucleosome alignments of read density (blue and red dots, respectively) given the four possible boundary conditions (both the +1 and −1 nucleosome may be directly or indirectly positioned) with nucleosome density and normalization being equal for both alignments (see ‘Materials and Methods’ for details). Regarding the mean squared deviation per data point, scenario C describes the data best, i.e., the scenario where the +1 nucleosome is directly positioned while the −1 nucleosome is indirectly positioned (Table S3). (1.00 MB PDF) [file pcbi.1000891.s005.pdf]

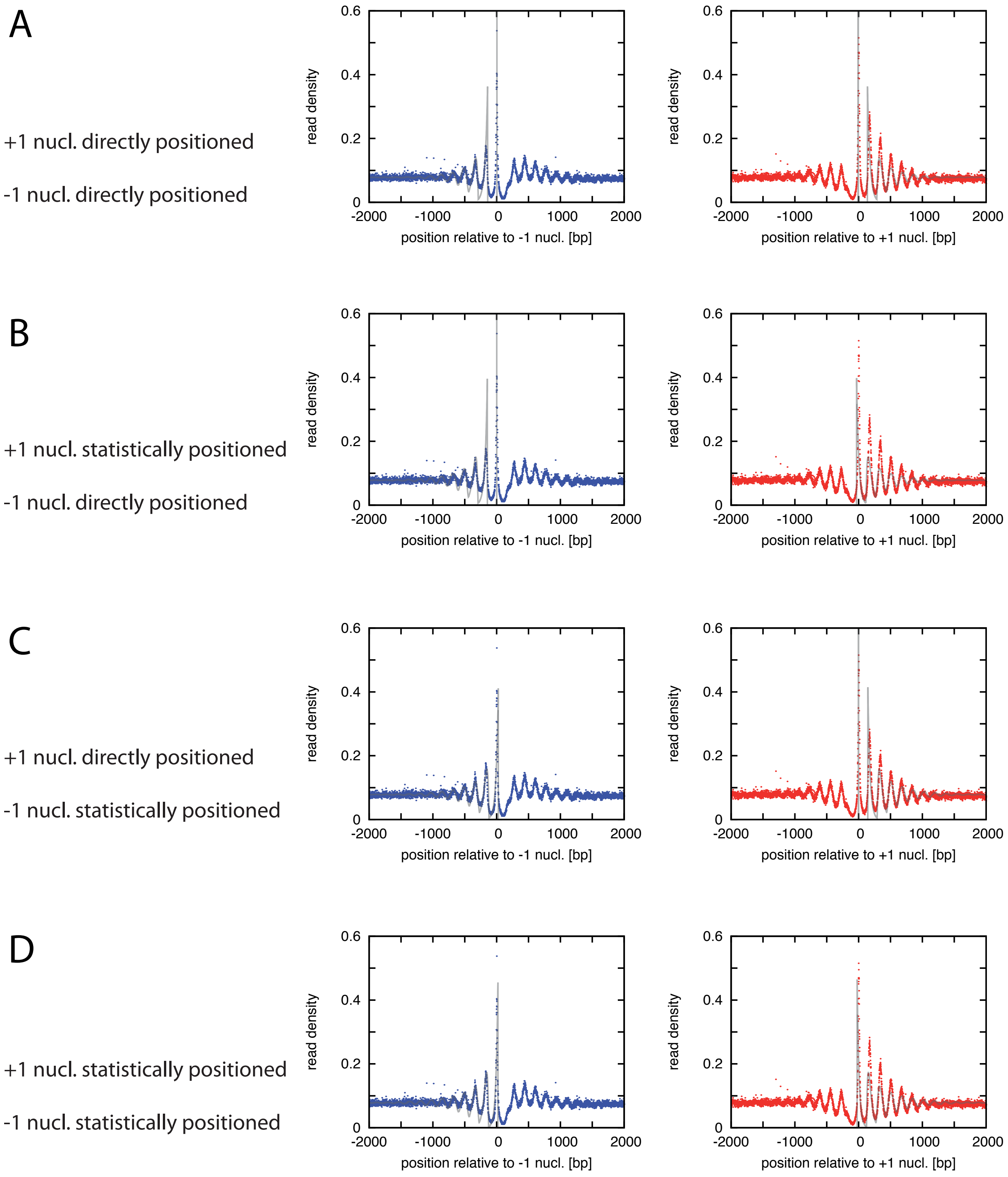
A
+1 nucl. directly positioned
-1 nucl. directly positioned
B
+1 nucl. statistically positioned
-1 nucl. directly positioned
C
+1 nucl. directly positioned
-1 nucl. statistically positioned
D
+1 nucl. statistically positioned
-1 nucl. statistically positioned
read density
position relative to -1 nucl. [bp]
position relative to +1 nucl. [bp]
0.6
0.4
0.2
0
-2000
-1000
0
1000
2000
