# Supplementary material for: Quantitative Test of the Barrier Nucleosome Model for Statistical Positioning of Nucleosomes Up- and Downstream of Transcription Start Sites
Source: PLoS Comput Biol. 2010 Aug 19;6(8):e1000891. doi: 10.1371/journal.pcbi.1000891 (PMC2924246; doi:10.1371/journal.pcbi.1000891)
Supplement: Table S1 — Parameter estimates from independent fits of Tonks gas model to nucleosome alignments of read density based on Equation (2) (density , normalization , offset , squared deviation per data point ). Numbers in parentheses indicate values that were set fixed rather than estimated from the fit. See ‘Materials and Methods’ for details. (0.03 MB PDF) [file pcbi.1000891.s007.pdf]

**Fit scenario**	$1/\bar{\rho}$ [bp]	λ	Δr [bp]	δ
+1 nucleosome, all genes, Fig. 2A	177	13.7	1	1.5e-4
+1 nucleosome, genes larger 2000 bp, Fig. S4	172	13.5	7	2.8e-4
+1 nucleosome, all genes, fit of convoluted funct., Fig. 2B	175	13.6	(0)	1.1e-4
-1 nucleosome, all genes, Fig. 4C	(177)	(13.7)	-169	9.8e-5
