# Supplementary material for: Quantitative Test of the Barrier Nucleosome Model for Statistical Positioning of Nucleosomes Up- and Downstream of Transcription Start Sites
Source: PLoS Comput Biol. 2010 Aug 19;6(8):e1000891. doi: 10.1371/journal.pcbi.1000891 (PMC2924246; doi:10.1371/journal.pcbi.1000891)
Supplement: Table S2 — Parameter estimates (density , normalization , offset , squared deviation per data point ) from fits of Tonks gas model to +1 nucleosome alignments of read density using subsets of genes only. Four times (partitioning A–D), the set of 4792 genes was divided into four equal-sized subsets (subset 1–4) before fitting. Estimated parameters are very similar; see ‘Materials and Methods’ for details. (0.03 MB PDF) [file pcbi.1000891.s008.pdf]

	$1/\overline{\rho}$ [bp]	λ	Δr [bp]	δ
Partitioning A, Subset 1	176.8	13.60	0.6	2.29e-4
Partitioning A, Subset 2	177.5	13.66	-0.4	2.15e-4
Partitioning A, Subset 3	176.9	13.65	1.3	2.46e-4
Partitioning A, Subset 4	177.5	13.74	0.6	2.45e-4
Partitioning B, Subset 1	176.6	13.79	1.6	2.30e-4
Partitioning B, Subset 2	176.6	13.65	0.6	2.54e-4
Partitioning B, Subset 3	177.9	13.53	-0.4	2.14e-4
Partitioning B, Subset 4	177.6	13.67	0.5	2.42e-4
Partitioning C, Subset 1	177.2	13.74	-0.3	2.36e-4
Partitioning C, Subset 2	176.6	13.60	0.7	2.31e-4
Partitioning C, Subset 3	177.9	13.74	0.6	2.48e-4
Partitioning C, Subset 4	177.3	13.59	0.6	2.24e-4
Partitioning D, Subset 1	176.9	13.65	1.4	2.26e-4
Partitioning D, Subset 2	177.3	13.76	0.4	2.39e-4
Partitioning D, Subset 3	177.2	13.61	-0.4	2.28e-4
Partitioning D, Subset 4	177.0	13.60	1.5	2.36e-4
