# Supplementary material for: Quantitative Test of the Barrier Nucleosome Model for Statistical Positioning of Nucleosomes Up- and Downstream of Transcription Start Sites
Source: PLoS Comput Biol. 2010 Aug 19;6(8):e1000891. doi: 10.1371/journal.pcbi.1000891 (PMC2924246; doi:10.1371/journal.pcbi.1000891)
Supplement: Table S3 — Parameter estimates for simultaneous fits of Tonks gas model to nucleosome alignments of read density. Both normalization and nucleosome density are constrained to be equal for both alignments. and are independent parameters accounting for different boundary conditions. Regarding the mean squared deviation per data point , scenario C describes the data best, i.e., the scenario where the +1 nucleosome is directly positioned while the −1 nucleosome is indirectly positioned (Fig. S5). See ‘Materials and Methods’ for details. (0.04 MB PDF) [file pcbi.1000891.s009.pdf]

**Fit scenario (see Fig. S5)**	$1/\overline{\rho}$ [bp]	λ	Δr_{-1} [bp]	Δr_{+1} [bp]	δ
scenario A	187	14.6	-6	-8	1.6e-4
scenario B	182	14.2	-2	-179	2.1e-4
scenario C	180	14.1	-174	-3	1.1e-4
scenario D	177	13.8	-168	-170	1.3e-4
